# Simulation and visualization of multiple KEGG pathways using BioNSi

**DOI:** 10.12688/f1000research.13254.2

**Published:** 2018-05-14

**Authors:** Adva Yeheskel, Adam Reiter, Metsada Pasmanik-Chor, Amir Rubinstein

**Affiliations:** 1 Bioinformatics unit, Faculty of Life Science, Tel Aviv University, Tel Aviv, Israel; 2 School of Computer Science, Tel Aviv University, Tel Aviv, Israel

**Keywords:** biological network, simulation, gene expression, KEGG, Cytoscape, BioNSi

## Abstract

**Motivation:** Many biologists are discouraged from using network simulation tools because these require manual, often tedious network construction. This situation calls for building new tools or extending existing ones with the ability to import biological pathways previously deposited in databases and analyze them, in order to produce novel biological insights at the pathway level.

**Results:** We have extended a network simulation tool (BioNSi), which now allows merging of multiple pathways from the KEGG pathway database into a single, coherent network, and visualizing its properties. Furthermore, the enhanced tool enables loading experimental expression data into the network and simulating its dynamics under various biological conditions or perturbations. As a proof of concept, we tested two sets of published experimental data, one related to inflammatory bowel disease condition and the other to breast cancer treatment. We predict some of the major observations obtained following these laboratory experiments, and provide new insights that may shed additional light on these results.

**Tool requirements: **Cytoscape 3.x, JAVA 8

**Availability:** The tool is freely available at http://bionsi.wix.com/bionsi, where a complete user guide and a step-by-step manual can also be found.

## Introduction

Modeling and simulation of biological regulatory networks is becoming an integral part of biological research nowadays ^1^. Such networks consist of nodes, representing, *e.g.,* proteins, RNA, genes, nutrients, signals, etc. Edges represent the interactions between nodes, such as activation, inhibition, phosphorylation, etc. Simulation tools allow the analysis and visualization of the dynamics of these networks. Biological data, such as protein interactions and gene or protein expression are gathered and provide the required input for such tools. Indeed, in recent years many bioinformatics tools aiming at the simulation of biological networks have been published. The purpose of such tools is to elucidate the relationships between genes, proteins, pathways, or other biological entities involved, to shed new light on the experimental results, and to suggest possible directions for future research.

Simulation tools come in various flavors. Some focus on transcriptional regulatory networks, others on signal propagation or metabolic pathways. There are tools that require programing skills, while others provide a complete graphical user interface (GUI). The underlying mathematical models differ a lot too. Perhaps the most fundamental distinction is between continuous, Boolean and discrete models. Continuous network models typically apply differential equations, using real (as opposed to discrete) numbers to represent the system’s variables ( *e.g.,* 2), while in Boolean network models variables may assume only one of two values, namely 0 (“OFF”, or *inactive*) and 1 (“ON”, or active) ( *e.g.,* 3). Between the two "extremes" are discrete models, in which values are taken from a range of integers ( *e.g.,* 0, 1, 2, …9). This is often a good compromise between the expressiveness of the model and its simplicity, and it is especially useful when only partial or imprecise biological knowledge of these systems is available ^4^.

Table 1 provides a summary of several network modeling and simulation tools properties. These tools are relatively new ones (most of them published within the last four years). In addition, we focused on biologist-friendly tools that require very little computational background and in particular no script writing skills. The last tool in Table 1 is BioNSi - Biological Network Simulator ^5^, whose extensions are the focus of this paper, as will be described later.

**Table 1.** A representative list of free, network simulation, tools for non-programming biologists. A representative list of seven network simulation tools was obtained from OmicsTools. A summary of some important features is shown. V: denotes that a specific feature exists in the tool. Web/App: a web tool or downloadable software. Nodes levels: Boolean (B)/discrete (D)/continuous (C). Non-simultaneous update: defining delays on edges or priorities on nodes, which inflict order on the update of nodes. Asynchronous update: non-deterministic behavior which enables several successors to a single network state. Batch simulation mode: running a collection of simulation with various parameters to reveal emergent network properties. Edges weights: weights represent strength of interaction. Network generation: textual (T)/graphical (G). A star (*) represents features implemented in the extended BioNSi tool (but not in the original version).

Tool (ref)	Web/App	Nodes levels	Non simultaneous update	Asynchronous update	Batch simulation mode	Edges weights	Network generation	Merge pathways	Upload expression data
**SimBoolNet** ^6^	app	C			V	V	T		
**GINsim** ^7^	app	D	V	V	V	V	G		
**SQUAD** ^8^	app	B+C			V		T+G		
**ViSiBooL** ^9^	app	B	V		V		T+G		
**ANIMO** ^10^	app+web	D	V				T+G		
**BooleSim** ^11^	web	B					T+G		
**BioNSi** ^5^	app	D	V		V	V	T+G	*	*

In Table 1 we compare the following features:

**–**    Web/App: whether the tool is web-based (web) or a stand-alone software or application (app)
**–**    Nodes' levels: the granularity of the model variables, that is, whether nodes levels are Boolean (B), discrete (D) or continuous (C) variables
**–**    Asynchronous update: whether the node level transition rules can be applied asynchronously, rather than simultaneously (for example, by specifying delays on edges, or defining priorities on edges)
**–**    Batch simulation mode: whether the tool supports a batch mode of simulation, in which multiple initial conditions are explored simultaneously and global (emergent) properties such as the system steady states (attractors) are identified
**–**    Edge weights: whether the edges in the network have weights to represent strength (amplitude) of interaction, in contrast to merely +1 (activation) and -1 (inhibition)
**–**    Network generation: which format is supported for network generation - graphical (G), in which the network is drawn on the canvas, or textual (T), in which the user generates a table of nodes and edges
**–**    Merge pathways: whether the tool supports merging of multiple pathways of interest into a single network.
**–**    Upload expression data: whether expression data from *e.g.,* laboratory experiments can be loaded into the network automatically.

Network simulation tools typically require manual network construction based on biological data. For networks beyond a very small scale of a few nodes, this process becomes tedious, time consuming, and error prone. Furthermore, conducting numerous simulations of a network under different biological conditions may require a tiresome and monotonous process of modifying network parameters for each set of conditions. This contributes to the discouragement of many biologists from integrating network simulation tools in their daily laboratory routine. This situation is extremely unfortunate, since a lot of network data based on lab experiments already exists in various common databases. Therefore, the integration of network simulation tools with biological data previously deposited into those databased provides novel opportunities for more comprehensive studies of these networks. Initiatives aiming at such integration may support biological understanding of previous laboratory results, and direct researchers to design new experiments.

There are several existing tools for the generation of mathematical models from KEGG pathway database information ^12^ (e.g., Büchel *et al*. ^13^, Wrzodek *et al*. ^14^). We aim at integrating a recently published Cytoscape application, BioNSi (Biological Network Simulator ^5^) with KEGG, exploiting this convenient simulation environment. For the sake of self-containment of this paper, Box 1 describes BioNSi at a high level. BioNSi was originally used for visualizing biological networks and simulating their time course behavior. A full user guide and a step by step manual can be found on the tool's website ( http://bionsi.wix.com/bionsi).

**Box 1. BioNSi - A high level description**

BioNSi is a biological network simulation tool ^5 ^. It allows constructing a regulatory network and simulating its behavior. The network consists of *nodes* (interactors) and *edges* (interactions). For example, nodes may represent genes, RNA or transcription factors, while edges represent interactions such as transcription, translation, phosphorylation, etc.

For each node, the user manually defines an initial expression level, termed *initial state*, an integer typically taken from the range between 0 and 9 (0 – no activity, 9 – high activity). Time in the model is also discrete, represented as clock "tics" or time steps ( *t* = 0, 1, 2,…). Nodes' states are updated in each time step by a transition rule (see below).

Each edge is assigned a weight which reflects the strength of the interaction between 2 nodes, and is either a positive integer (activation, translation, etc.) or a negative one (repression, inhibition, degradation, etc.). Degradation usually appears as a self-negative edge. The effect of node *i* on node *j* is the product of node *i*'s state and the weight of the edge *i*→ *j*. Thus, the strength of this effect is proportional to both the weight of the edge and the relative level of activity of the source node. The "net" effect on a node is the sum of effects of all nodes on it. Figure A shows a "toy" network with 4 nodes (A, B, C, signal; colored rectangles), whose initial states are shown below their names. Edges are labeled with their weights and colored accordingly (green = activation; red = inhibition).

**Figure:**
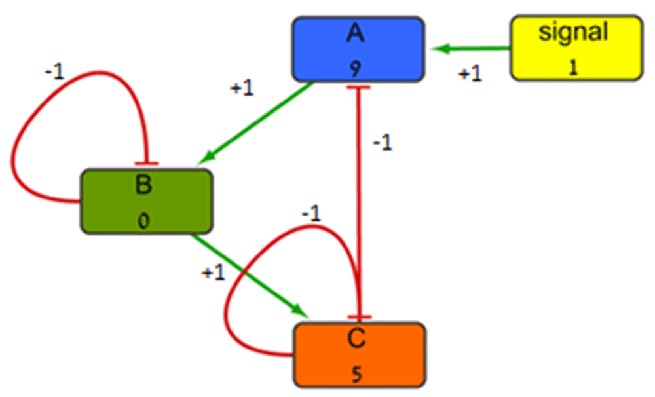
Figure A: a "toy" network in BioNSi

**Figure:**
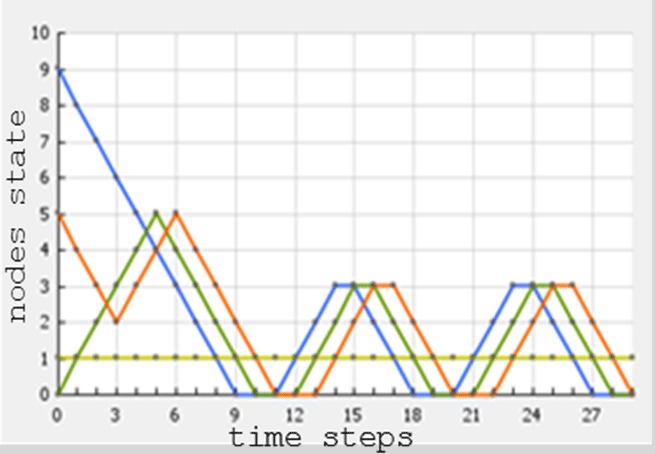
Figure B: graphical presentation of simulation results

A simulation starts with a given configuration of initial states, and consists of the repeated application of a transition rule, simultaneously to all nodes: the state of a node will increase, decrease, or remain unchanged when the "net" effect on it is, respectively, positive, negative or 0. The simulation ends when either a steady state is reached (two consecutive identical configurations of nodes' states), or a loop of configurations is detected.

The tool presents the course of simulation graphically (see Figure B). The network exhibits an infinite loop in which the states of nodes A, B and C repeatedly go up and down, while the signal node is constant at state 1. BioNSi also enables a batch mode, which is used to gather statistics on a set of simulations, to study global properties of the network ( *e.g.*, the distribution of steady states, average time to get to each steady state, etc.).

BioNSi supports various additional features, which are described in the manual on the website. These include (i) delay on edges for non-simultaneous update of nodes, (ii) blocking edges, where nodes X1, X2, … can block the effect of some node Y on node Z at a specific time step. The blocking nodes can act together or each of them separately. And (iii) nodes that represent external signals (hormone injection, sunlight, etc.) with a pre-defined expression pattern whose behavior is pre-set by the user, etc.

**Figure 1.**
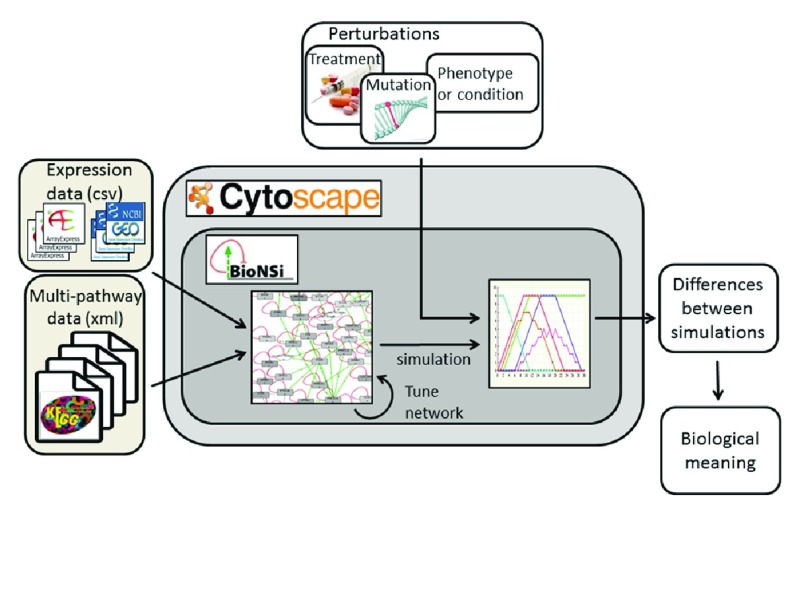
The extended BioNSi simulation tool. Gene expression data and selected KEGG pathway data-files are uploaded to BioNSi’s Cytoscape Application and analyzed. The resulting network can be modified and tuned. Simulation is performed for gene expression data resulting from various conditions, possibly with addition of artificial perturbations. Differences in the results of simulation under various conditions provide novel biological insights.

Our new developments enable merging of multiple KEGG pathways into a single coherent network under the BioNSi framework. Although the files downloaded from KEGG may omit some protein-protein interactions details, they were found to be useful in presenting networks based on the researcher’s interest. Furthermore, uploading experimental expression data enables conducting a simulation of the network. The initial expression levels of network nodes (genes, proteins, etc.) may be obtained either from in-house experiments or from various expression databases ( *e.g.,*, GEO ^15^ or ArrayExpress ^16^. This allows easy simulation of the resulting network under different experimental conditions (expression values). For example, one can simulate the effect of inhibitors, activators, mutations, abnormal cell conditions or the effect of a new drug on the network's behavior, suggesting further experimental exploration. BioNSi is a Cytoscape application ^17^. Cytoscape is an open source bioinformatics software platform, which hosts various applications for visualization and analysis of networks. The benefit of this is that the BioNSi simulation results can be further extended with additional analyses based on other applications. The typical workflow with the new version of BioNSi is presented in Figure 1.

We first describe the details of our new extensions to BioNSi. Then, as a proof of concept, we demonstrate the advantages and usability of our tool by presenting the simulation and analysis of previously published experimental data from two sets of experiments: (1) the characterization of inflammatory bowel disease (IBD) conditions, and (2) treatment of MCF7 breast cancer cells with heregulin (HRG). These two test cases may provide insights into how our tool can be used to elucidate existing biological data, raise new hypotheses and suggest new lab experiments to test them.

## Methods

### Implementation

Cytoscape is an open source software platform for complex network analyses and visualization ^17^. It enables "plugging in" in the form of dedicated software called applications (Apps), which are developed by users. BioNSi ^5 ^is one such application that provides a user friendly tool for simulating the dynamics of biological regulatory networks. It is implemented under Cytoscape version 3. Installation is easy and explained in the tool's website at http://bionsi.wix.com/bionsi. We have extended BioNSi’s capabilities with two key features presented in the next sections.

### Operation

***Importing and merging multiple KEGG pathways into BioNSi.*** The original version of BioNSi allowed creating networks “ *de novo*”, by adding nodes and edges manually, according to the biological knowledge of the researcher. This may be a tedious task, when the networks are beyond a very small size. The new feature allows easily importing multiple pathway files that were exported from the KEGG database. The pathways are simply downloaded from KEGG as xml files, and merged into a single, integrated network under BioNSi. The user guide on BioNSi's website explains the details for this operation. Briefly, nodes contained in several entities often have alternative pathways are fused into a single node, with all their incoming and outgoing edges. KEGG names are all preserved for each node to allow matching of the same entity with different KEGG name.

The user can specify the desired weights for each KEGG type of edge, such as activation, inhibition, phosphorylation, etc., or rely on the default values provided. As biological materials are routinely being degraded, "self-inhibition" loops are automatically added to each node. Subsequently, the resulting network can be "tuned" by adding or removing specific nodes and edges, or by changing specific parameters ( *e.g.,* the weight of a specific edge). Thus, constructing large-scale integrative networks from known pathways is done with little effort, and enables inspecting biological pathways at a broader biological context.

***Importing nodes’ initial states.*** The second new feature allows the initial nodes' states obtained either from in-house experiments or from various expression databases to be loaded from a simple text file (a two-column comma separated values (csv) text file, in which the left column contains nodes' names and the right one contains the desired initial levels). Such a file can be easily generated from most existing gene expression databases, using *e.g.,* Microsoft Excel. Since existing expression data sources may have different scales ( *e.g.,* logarithmic vs. linear), nodes' initial states are read from the input file and normalized to the range 0–9 for consistency and convenience. Note that the initial states are scaled to 0–9 for the whole network based on the maximal expression level in the network, rather than for the range of each gene separately. Since the scaling depends of the maximal expression level of a node in the network, an extreme outlier may restrict the levels of other nodes to a very limited range. Therefore, the use of logarithmic expression data is recommended.

In addition, we extended BioNSi with a graphical representation of the nodes' levels: nodes' background colors indicate their initial states (higher levels are darker gray). Once a simulation ends, nodes colors are automatically updated to reflect their final state, providing a visual image of the incline or decline in nodes' expression. This new feature allows easy comparison between the network's behavior under different experimental conditions, by loading alternative sets of initial states and conducting a new simulation each time.

***Simulation.*** We explored two use-cases as a proof of concept, as described in the next section. In both use cases, KEGG pathways were integrated to form a BioNSi network, and gene expression analysis was used as initial nodes' states. In both use cases, the states of nodes changed throughout the simulation process, terminating in a steady state. This change in states may reflect one of two situations: (i) the expression data itself does not represent a biological steady state, and therefore the changes *in-silico* reflect changes *in-vivo*, and (ii) data is missing from the network, either structural (missing regulators or connections between existing nodes) or quantitative (weights on edges, initial states, etc.). In the latter case the conclusion is that the model needs further improvement.

In the two test cases described in the *Use Cases* section, we conducted simulations for 100 time-steps. All simulations reached a steady state within this number of steps. In order to compare between simulation results, we had to define when a node is considered to exhibit different behavior in the two simulations. We used a simple Euclidian distance measure between the two simulation trajectories of each node. Furthermore, we filtered out nodes whose difference in the two simulations is merely a result of a different initial state. We did that by considering only nodes for which the difference between the simulations, at some time step, was larger than the difference in the initial state. In other words, the gap between the node's states in the two compared simulations should increase at some point beyond the initial gap, in order for it to be considered differential between the simulations. An implementation of this distance function can be found as a Python script (diff.py) that can be downloaded from the tool's website.

## Use cases

### Case study 1: Characterization of inflammatory bowel disease (IBD) conditions

IBD are a series of chronic inflammations of the intestine. Pouch surgery is a useful treatment of IBD patients, but pouch inflammation is a very common outcome ^18^. Crohn’s-like disease of the pouch (CLDP) is the most severe inflammation condition. Activation of NFKB was identified as one of the key regulators inducing IBD inflammation by promoting pro-inflammatory cytokines ^19, 20^. Parthenolide, a strong NFKB inhibitor, has recently been demonstrated to be a promising therapeutic agent in IBD inflammation, promoting apoptosis of cancer cells ^21^. We aim at extending the original study analyzing differentially expressed genes and their functional enrichment, to suggest new targets for treatment.

***Expression data source.*** Affymetrix GeneChip (Human Gene 1.0 ST arrays) was used for gene expression analysis of normal and CLDP donors, as previously described ^18^.

***Pathway data.*** As reported in 18, 74 genes were found to be up-regulated in CLDP vs normal expression (pFDR<0.05 and fold-change difference=5). WebGestalt KEGG pathway enrichment ^22^ revealed enriched 16 pathways (pBonf<0.05), of which the top 5 were selected for analysis ( Table 2). In addition, IBD pathway was added, although not specifically enriched in the analysis (total of 6 pathways).

**Table 2.** Five top pathways enriched (adjP) in differentially expressed gene analysis ^16^, in addition to inflammatory bowel diseases (IBD) pathway. Top human KEGG pathways were obtained and enrichment p-values presented. Links to KEGG xml file download are provided.

KEGG enrichment	KEGG (hsa)	adjP	Link to xml files
**Cytokine-cytokine receptor** **interaction**	04060	1.50e-11	http://www.genome.jp/kegg-bin/show_pathway?hsa04060
**Leishmaniasis**	05140	6.52e-10	http://www.genome.jp/kegg-bin/show_pathway?hsa05140
**Rheumatoid arthritis**	05323	3.49e-09	http://www.genome.jp/kegg-bin/show_pathway?hsa05323
**Chemokine signaling pathway**	04062	1.98e-08	http://www.genome.jp/kegg-bin/show_pathway?hsa04062
**Hematopoietic cell lineage**	04640	7.13e-06	http://www.genome.jp/kegg-bin/show_pathway?hsa04640
**IBD**	05321	Not enriched	http://www.genome.jp/kegg-bin/show_pathway?hsa05321

***NetworkAnalyzer out-degree analysis.*** Network analysis was performed using NetworkAnalyzer tool, which is another built-in Cytoscape application ^23^. We used it in order to visualize the most out-connected (notably high out-degree) genes in the network. The most outstanding such node is NFKB1 (19 outgoing edges, see Table 3). Therefore, an additional NFKB signaling pathway ( hsa04064) was added from KEGG. Altogether, 7 KEGG pathways were analyzed, composed of 379 nodes (mostly genes).

**Table 3.** Node distribution by out degree score. Cytoscape’s NetworkAnalyzer tool was used to present out-degree score. NFKB1 has an outstanding out-degree score.

**Out-degree**	0	1	2	3	4	5	6	7	8	9	10	11	12	13	14	15	16	17	18	19
**Number of nodes**	0	138	155	51	18	11	2	3	0	0	0	0	0	0	0	0	0	0	0	1 (NFKB1)

**Figure 2.**
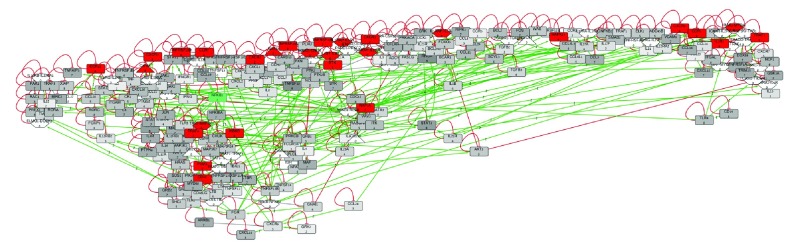
The main component of the CLDP test case network. We show only 241 out of 379 genes, which belong to the main connected subnetwork resulting from 7 KEGG pathways (excluding subnetworks of 8 nodes or less). We used Cytoscape’s hierarchical layout, in which nodes with no outgoing edges appear at the top. Gene’s fill color is according to control expression values (grayscale), except nodes that are different between the simulation of CLDP and control expression, which are marked in red, and NFKB1 which is colored turquoise. Edges are colored according to type of interaction (activation–green; inhibition-red). For convenience, genes or proteins are represented as rectangles, compounds as triangles and complexes (dimers) are hexagons.

In the resulting merged network ( Figure 2), in addition to genes, compounds and complexes were also detected (presented as triangles and hexagons, respectively). These were automatically assigned expression value 0 as no information could be found for them in the expression data.

***Edge weights.*** We used edge weights as following: inhibition -1, activation +2. As biological materials are being degraded over time, self-inhibition edges (weight -1) were automatically added for each node in the network. Accordingly, dephosphorylation (-p) edges are set to -1. Activation edges, including phosphorylation (+p) were set to +2, to compensate for the degradation edges automatically added to all nodes.

***Results.*** We aim at comparing between two simulations: 1) Control gene expression; 2) CLDP gene expression. The network consists of 379 nodes. The comparison revealed 49 nodes that exhibited different simulation trajectories (genes, compounds and complexes; 12.9% of all nodes in network). 87.8% of these (43 nodes) were downstream in the network, namely, had no outgoing edges (except for self-loops that all nodes have). To visualize this we used Cytoscape’s hierarchical layout, as shown in Figure 2. The nodes that differed between the two simulations are colored red. The hierarchical layout positions these nodes at the top of the network. NFKB1 gene (highlighted in turquoise) is highly connected within the network, but not to any of the selected (red) genes.

A large fraction of the network’s genes is not directly connected to the main network, but forms small independent sub-networks (138 nodes with 8 nodes or less). This is a result of selecting only 7 KEGG pathways for the analysis. Naturally, the analysis is highly dependent on the KEGG pathways selected. Disconnected sub-networks may be connected to the main network following the inclusion of additional, less enriched KEGG pathways. However, these subnetworks follow the same pattern seen in the main network, and all of the nodes differing in the 2 simulations are at the top of the hierarchy ( Supplement 1; http://www.cs.tau.ac.il/~amirr/files/supp/testcase1_files.zip). For clarity, Figure 2 shows only the main connected subnetwork (241 nodes; 138 nodes were removed from Figure 2, all harboring connected subnetworks with 8 or less nodes, presented in Supplement 1).

In terms of biological function, most of the 49 nodes that exhibit differences between the simulations are of genes that are either immune system receptors (receptors for chemokines, cytokines, interferons, interleukins, PDGF, growth factors (MET) etc.), or various kinases (BTK, MAPK14, MAP3K7, IKBKB, FLT3 and FLT4). Many therapeutic agents are offered based on such molecules ^24^. Several cytokines (that may be targeted by JAK inhibition), were found within the BioNSi resulting nodes that exhibited different simulation trajectories: IL6ST, IL6R and OSMR (gp130 family member), IL10RB (IL10 members), IL2RB, IL7R (γc family) and CSF1R (βc family), and IFNAR1, IFNAR2 (interferon related genes) ^25^. This analysis may suggest interesting new targets for drug response.

### Case study 2: Treatment of MCF7 breast cancer cells with heregulin (HRG)

The purpose of the following example is to demonstrate the effect of HRG treatment on MCF7 breast cancer cells, based on published GEO experimental results ( GSE6462; ^24, 25^. The simulation results suggest that BioNSi is capable of recapitulating the major changes in the network after drug treatment, as observed by *in vitro* experiment.

***Expression data source.*** MCF7 breast cancer cells were treated with a growth hormone, HRG, at four different doses and different time course (GSE6462). We used expression data after maximal treatment of 10 nM HRG for 90 minutes. In the experiment, the mRNA expression levels were measured for control (GSM148517) and HRG treated (GSM148572) cells (using Affymetrix Human Genome U133A 2.0 Arrays).

***Pathway data.*** KEGG-xml files for three selected human pathways were exported from the KEGG database ^12^: ERBB signaling pathway ( hsa04012); MAPK signaling pathway ( hsa04010); EGFR cytosine kinase inhibitor resistance pathway ( hsa01521). These three representative pathways were reported to be crucial for the HRG treatment response ^26^. The merged network based on these three KEGG pathways consisted of 190 nodes.

***Setting network parameters and post-import modification.*** We used edge weights as follows: inhibition -1, activation +2. As biological materials are being degraded over time, self-inhibition edges (weight -1) were automatically added for each node in the network. Accordingly, dephosphorylation (-p) edges are set to -1. Activation edges (including phosphorylation; +p) were set to +2, to compensate for the degradation edges automatically added to all nodes. A node representing HRG treatment was added manually (diamond shape), either with initial state 0 (without drug), or with initial state 9 (active drug). The HRG node was directly connected to its known target nodes, ErbB3 and ErbB4, in addition to their homodimers nodes, with strong inhibition edges (weight -10 each). Protein homodimers (resulting from KEGG database) were given expression values similar to their monomer molecules (from the microarray mRNA expression results). Heterodimers were given initial state 0, as we have no data concerning their actual expression.

**Figure 3.**
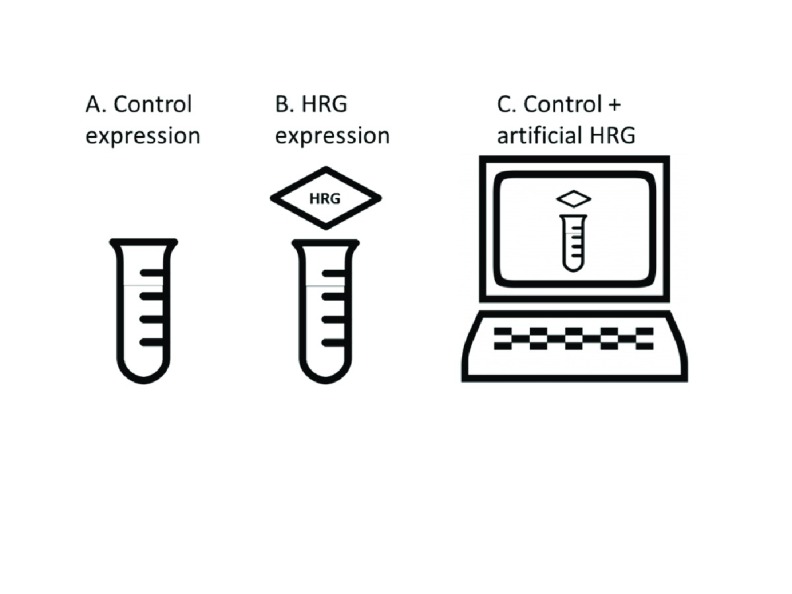
Schematic illustration of BioNSi simulations performed. Expression data from GSE6462 experiment was obtained for Control MCF7 breast cancer cells, without ( **A**) or with ( **B**) HRG treatment. ( **C**) BioNSi simulation of artificial HRG effect performed on control expression.

***Simulations.*** A useful application of the BioNSi tool is to compare between the simulation results under different nodes' initial states (reflecting different biological conditions). As illustrated in Figure 3, we compared simulations of control (A) vs HRG treatment (B) gene expression, in addition to simulation of artificial HRG effect on control gene expression (C).

In order to simulate artificial HRG treatment effects on the network, we used control expression values as initial states, and the HRG node at initial state 9. Network’s simulation under these conditions resulted in a complete and rapid decline of ErbB3, ErbB4 and their homodimer expression values.

**Figure 4.**
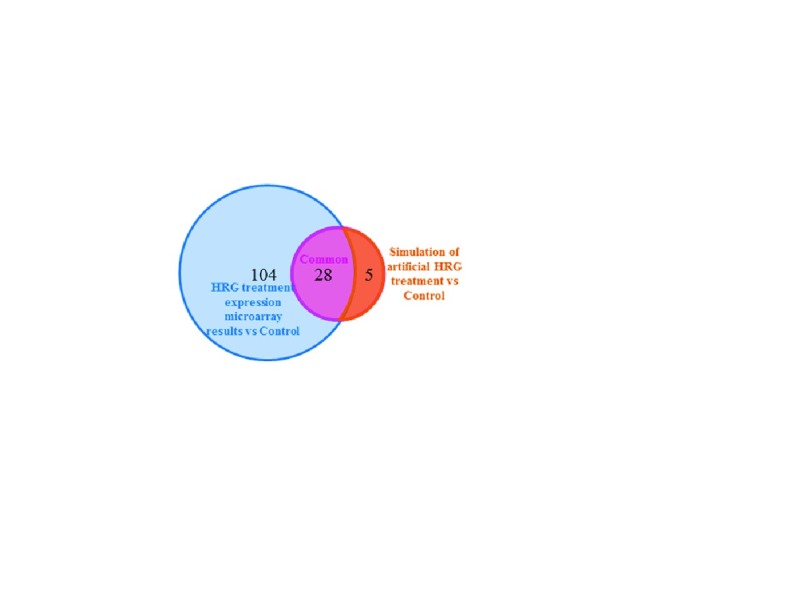
Venn diagram comparing simulation of HRG expression microarray results with simulation of artificial HRG treatment prediction. 28 genes were common to both analyses (pink). 5 genes were unique to simulation (orange), and 104 genes were unique to experimental analysis (blue).

***Results.*** We aimed at comparing between two simulation sets, based on the same network created: (1) Simulation that compares between HRG gene expression vs. control gene expression. The comparison between these simulations revealed 132 different genes (69.5% of all genes in the network (190)) whose expression pattern differs between the simulations of HRG vs Control. (2) Simulation of HRG artificial treatment effect on the same control gene expression, vs. control expression. This comparison revealed 33 genes (17% of the total 190 in network) that were found to be different. Figure 4 shows a Venn diagram that demonstrates these findings. 28 genes are common to both analyses. Artificial HRG treatment results (28 genes) represent ~21% of the experimental results (132 genes). 5 nodes were found to be uniquely changed by the prediction analysis, including the obviously changed (ERBB3, ERBB4 and ERBB4-ERBB4, which are directly inhibited by HRG simulation prediction, in addition to STAT5A).

**Figure 5.**
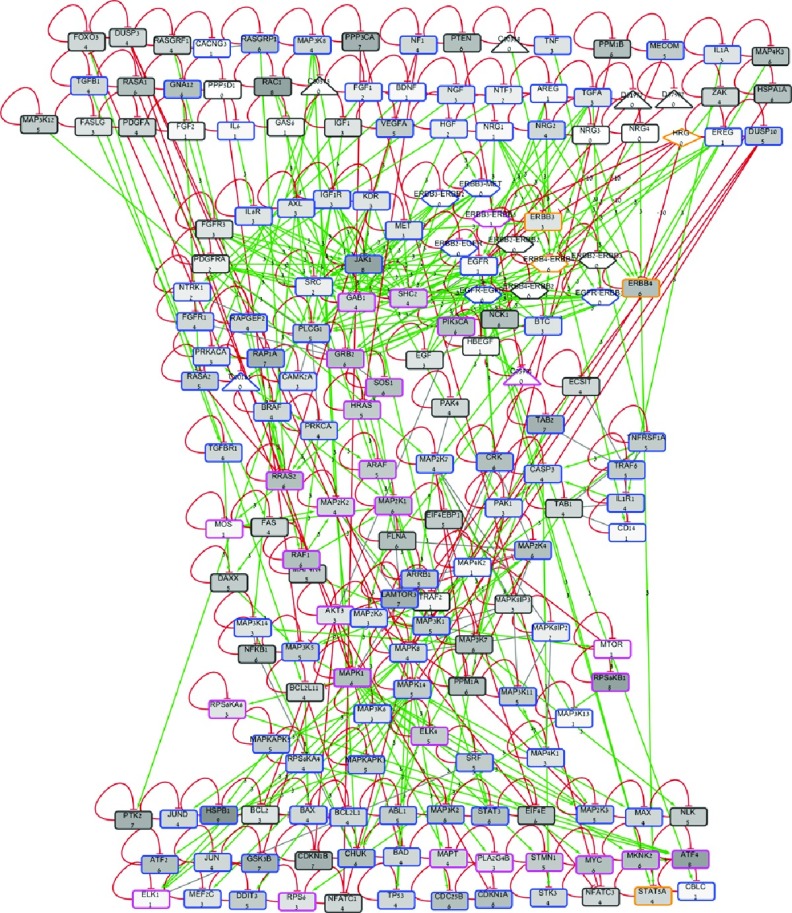
HRG network view. A network of 190 genes consisting of 3 KEGG pathways (described in the text), using control expression values is shown. Gene frames color code: common genes to the two analyses (pink), unique to prediction of artificial HRG treatment vs control (orange), unique to simulation resulting from HRG treatment vs control expression analysis (blue) or unchanged (black). The network shows genes with nodes fill color grayscale according to expression values and edge colors according to type of interaction (activation–green; inhibition-red). For convenience, genes or proteins are represented as rectangles, compounds as triangles and complexes (dimers) are hexagons. HRG treatment is presented as orange-framed diamond.

Interesting observations concerning the resulting genes can be observed in the BioNSi screenshot of the network in Figure 5. All the 33 genes (framed pink and orange), resulting from the artificial HRG treatment simulation analysis compared to control, are highly connected, especially downstream of the network. This suggests an interesting signal path of the HRG drug response, which may offer new targets for treatment. We note that the PI3K/AKT/mTOR and STAT pathway genes are predominantly changed following HRG artificial treatment prediction, pathways that may affect cell proliferation and apoptosis, as recently suggested ^5, 26^.

## Conclusions

The bio-technological revolution we are witnessing in recent decades enables generating unprecedented amounts of biological data of various types. This includes expression levels of RNA and proteins, and the interactions between them and other substances, such as nutrients, hormones, external stimuli etc. While these data are accessible to researchers, their analyses at the system level are less so. Extracting specific and local information is easy, but performing large scale network analyses requires computational approaches, such as simulation tools. However, existing network simulation tools are often meant for manual construction by the researcher of the network under study, and therefore remain inefficient for studying networks beyond a small scale. This is extremely unfortunate in an era when computational approaches to study biological systems are proliferating. Network simulations are one such important computational approach, which has proven efficient in providing new insights, shedding light on existing results, and suggesting new directions for exploration, both in the lab and on the computer. In order for simulation tools to become an integral part of the researchers' lab routine, tools that enable simulation of large scale network data are called for. Such tools must avoid a tedious network construction process, and allow a high level representation of networks to be generated from existing data semi-automatically with little effort and no computational skills.

In this paper we suggest an extension to an existing network simulation tool, BioNSi, that allows researchers to import multiple pathway data from the KEGG database into a single network representation. The tool then allows for the study of the network via simulations, under different biological conditions. We believe such an approach will foster the use of computational tools in the lab by researchers with little or no computational background.

## Software and data availability

Tool requirements: Cytoscape 3.x, JAVA 8

Tool download: http://bionsi.wix.com/bionsi (a full user guide and a step by step manual can be found at the tool's website)

Tool source code: http://doi.org/10.5281/zenodo.1065352 ^27^

Archived source code as at time of publication: http://doi.org/10.5281/zenodo.1065352 ^27^

License: CC BY 4.0

Data source for test case 1: https://www.ncbi.nlm.nih.gov/pubmed/24108111

Data source for test case 2: ftp://ftp.ncbi.nlm.nih.gov/geo/series/GSE6nnn/GSE6462/matrix/GSE6462_series_matrix.txt.gz

## Supplementary material

Test case 1 files: http://www.cs.tau.ac.il/~amirr/files/supp/testcase1_files.zip

Test case 2 files: http://www.cs.tau.ac.il/~amirr/files/supp/testcase2_files.zip
