# Supplementary material for: Simulation and visualization of multiple KEGG pathways using BioNSi
Source: F1000Res. 2018 May 14;6:2120. Originally published 2017 Dec 11. [Version 2] doi: 10.12688/f1000research.13254.2 (PMC6008849; doi:10.12688/f1000research.13254.2)
Supplement: Peer review history

# Peer review: Referee response for version 2

**DOI:** 10.5256/f1000research.16110.r34005

**Published:** 2018-06-14

**Authors:** Ester Feldmesser, Bareket Dassa

**Affiliations:** 1 Life Sciences Core Facilities , Weizmann Institute of Science, Rehovot, Israel; 2 Life Sciences Core Facilities, Weizmann Institute of Science, Rehovot, Israel

We believe that the extension tools for BioNSi, which are presented in the paper, provide useful network tools for biologists. Still, we suggest several improvements that are listed below.

1. The feature to compare between two networks is useful for the user, however more detailed explanations on how to work with it and how to visualize the differences between networks are missing. We would like to suggest that this feature will be integrated in the BioNSi application.
2. We would like to suggest that manual changes in the node states in the visualized network will affect the resulting node color in the simulation.
3. Uploading an expression data file with negative expression values (log2) is currently not applicable and should be corrected.
4. The names of the edges in the “Edge Table” panel are not informative and it is difficult to relate between the graphics and the table. In the example “toy1” network, there are two edges with the name “Edge_0” and one with “Edge_1”.
5. The option to “Destroy Network” exists in two places in the top menu: in the “BioNSi” tab and in the “Edit” tab. Please note that it worked properly only when activated from the “BioNSi” tab.

We have read this submission. We believe that we have an appropriate level of expertise to confirm that it is of an acceptable scientific standard.

# Peer review: Referee response for version 2

**DOI:** 10.5256/f1000research.16110.r33996

**Published:** 2018-06-11

**Authors:** Aurélien Naldi

**Affiliations:** 1 École Normale Supérieure (ENS), Paris, France

The revised article answered most of my reservations with the first version, some minor clarifications are still needed.

 The sentence explaining the merging process of multiple pathways is not clear and seems to merge two different sentences: "nodes contained in several entities often have alternative pathways are fused into  single node". What happens if gene A is associated to genes B and C in one pathway and to genes C and D in another? Will we obtain:

- a single node merging A,B,C, and D?
- a node merging A and C, and separate nodes for B and D?
- 4 different nodes?

I assume the authors selected the later solution, which facilitates the mapping of external data (as each node corresponds to a single biological entity), but increases the complexity of the network. Is that correct?

 Regarding the mapping of external data, I understand from the answer of the authors that the provided csv file will map official gene symbols, HGNC IDS, as well as KEGG-IDs. It is clearly stated in the documentation, but could deserve a comment in the paper, along with the fact that unmapped nodes will be assigned the value 0.

 While the misleading use if asynchronous in table 1 has been fixed, the text referring to this table in the introduction has not been updated.

 A closing parenthesis is missing on page 4: "(e.g. GEO or ArrayExpress".

I have read this submission. I believe that I have an appropriate level of expertise to confirm that it is of an acceptable scientific standard.

# Peer review: Referee response for version 1

**DOI:** 10.5256/f1000research.14379.r29782

**Published:** 2018-02-21

**Authors:** Giovanni Scardoni

**Affiliations:** 1 Center for Biomedical Computing (CBMC), University of Verona, Verona, Italy

I tried to simulate the toy example from the BioNSi website, following the step by step guide.

 It doesn't work. Tried some other examples (cellcyclesB, Testcase1_network) and no results.

 Maybe there is a bug (I am using Cytoscape 3.6.0) or there is something I'm missing. In the second case the step by step guide should be improved.

 This doesn't allow me to provide a complete evaluation of the tool and the paper that seems interesting and well written. I will be happy to reconsider my review once such problems are solved.

I have read this submission. I believe that I have an appropriate level of expertise to confirm that it is of an acceptable scientific standard, however I have significant reservations, as outlined above.

# Response

**Published:** 2018-03-06

**Authors:** Amir Rubinstein

**Affiliations:** Tel Aviv University, Israel

Dear Giovanni,

​Thank you for the prompt feedback.

After carefully reviewing the data again, we found a bug in opening the toy.cys network on some combinations of Cytoscape, Java and operating systems. We are working on fixing it. In the meantime, we double checked and the csv format should work properly on all versions. 

Therefore, we temporarily removed the cys files from the examples tab, leaving only the csv files. In addition, we slightly changed the step-by-step pdf manual to match these changes.

We hope this will allow a complete evaluation of the new version of BioNsi.

Thank you,

Amir

# Peer review: Referee response for version 1

**DOI:** 10.5256/f1000research.14379.r29573

**Published:** 2018-02-05

**Authors:** Aurélien Naldi

**Affiliations:** 1 École Normale Supérieure (ENS), Paris, France

This paper presents an extension of the BioNSi modeling software with two aims: (i) kickstart model construction by integrating KEGG pathways and (ii) use experimental expression data as initial states for the simulation. These aims make sense: facilitating the initial construction and evaluation of dynamical models is an important challenge in systems biology. However, I feel that details on the inner working and discussion of the limitations of this tool are missing from the paper. Some related work should also be taken into account. In particular Wrzodek et al. ^1^ and Büchel et al. ^2^ which generate qualitative models in SBML qual format ^3^ from KEGG pathways. My main concerns are detailed in the following text, none of them would be blocking taken separately, but my overall interpretation is that this work requires deep changes to become convincing.

 I installed the BioNSi tool in Cytoscape 3.4. The documentation states that building a network from KEGG pathways requires the selection of a folder containing one or several KEGG xml files and a CSV file with expression data. I tried to provide a folder containing two KEGG pathways and an empty CSV file, but it did not work (error message: "number of expression files is not 1"). I had the same problem with Cytoscape 3.6.

 KEGG pathways often "merge" multiple biological entities in a single node, the paper does not say how this case is handled, the documentation only states that ellipsis dots are removed from the name. Does it mean that these groups are preserved? How does this affect merging multiple KEGG pathways? It can be a concern as the same entity could be involved in different groupings in different KEGG pathways.

 More generally, it would make sense to separate the transformation of KEGG pathways into discrete models (taking into account previous work ^1^^,^^2^), and the merging of multiple discrete models, which is a more general problem, and could be used to integrate several existing models with new models generated from pathway maps.

 The import of initial states from experimental data, and in particular the discretization step deserve further discussion. For a generic tool, I suspect that it uses a simple method where the full range of values for all genes is scaled to 0-9, defining 9 evenly-spaced thresholds. Is it the case or are the thresholds based on the range of each gene separately? As mapping experimental data on the model is one of the two core aims of this paper, it should at least review the alternatives, give proper details on how this is handled and state that alternative mapping can be integrated as a preprocessing step.

 Mapping entities in the model to entities in the experimental data is not discussed either, does the tool convert KEGG identifiers to official gene names, does it require the user to do the mapping in advance, does it provide a GUI to fine-tune the mapping afterwards?

 The paper does not state what is the objective of using an experimentally measured values as initial state. The experiments presented here seem to represent "steady states" of the biological system, the simulation leads to a steady state which can be compared to the experimentally measured one. Differences between the experimental and computed states can be of two types: (i) inconsistency showing that the model needs further improvement, and (ii) effect that is not measured in the experiment: for example change of phosphorylation which is not measured by expression data. As this is the very core of the paper, a discussion on the possible biological meaning of the results seems required.

 The BioNSi tool, which this work extends, supports simulations of qualitative (discrete) models. Its capabilities are summarized in this paper, and it is briefly compared to other tools for qualitative modelling in Table 1. A minor typo in this table replaced BooleSim with BoolSim (there is also a tool called BoolSim in this field: it is the command line tool handling the Boolean part of SQUAD). The paper describes BioNSi (and some other tools) as doing synchronous and asynchronous simulations, but misuses the term "asynchronous". Here asynchronous refers to synchronous with delays or priorities (i.e. all transitions do not happen at the same speed, but are still using the same clock), however it usually means independent trajectories, leading to non-deterministic behaviour. Using the classical asynchronous updating of qualitative models, a state can have several separate successors, each corresponding to the update of a single component. This allows to study systems where the relative speeds of transitions is not known, but introduces numerous concurrent trajectories, making the analysis much harder. GINsim is the only tool in the provided list implementing asynchronous updating, it also supports priorities, but not delays. BoolNet ^4^, PyBoolNet ^5^, and Boolsim ^6^ support asynchronous updating. To handle this complexity, some tools such as BooleanNet ^7^ or MaBoSS ^8^ perform random walk in the asynchronous dynamics.

 In BioNSi, inhibitions have negative weights, while activations have positive weights. These weights are multiplied by the level of their source nodes, the resulting weighted sum is then used to define the evolution of the target element. While this allows to quickly define a model, I am concerned by the lack of flexibility: some inhibitors only block the effect of one of the activators, some regulators have a cooperative effect, some act independently. I agree that the simple weight system used in BioNSi is convenient when these fine-grained mechanisms are unknown: it provides a good first model. However, the KEGG pathways mostly describe processes which have been deeply studied, and for which such an approximation could be avoided. There is some value in a simple system based on weights, especially as it fits better for integration into cytoscape, but building more precise functions through the use of intermediate nodes representing cooperative effects should at least be discussed as a common refinement.

 Some minor points:

- Missing closing of 2 parenthesis on page 2, another on page 3
- Page 6: "138 nodes with 8 nodes or less" is confusing. I suspect it means 138 nodes within sub-networks of 8 nodes or less?
- The first paragraph on page 6 ("simulation of different experimental conditions") repeats the last paragraph of the methods.

I have read this submission. I believe that I have an appropriate level of expertise to state that I do not consider it to be of an acceptable scientific standard, for reasons outlined above.

## References

[1] WrzodekCBüchelFRuffMDrägerAZellA: Precise generation of systems biology models from KEGG pathways. *BMC Syst Biol*.2013;7: 10.1186/1752-0509-7-15 15 10.1186/1752-0509-7-15 23433509PMC3623889

[2] BüchelFRodriguezNSwainstonNWrzodekCCzaudernaTKellerRMittagFSchubertMGlontMGolebiewskiMvan IerselMKeatingSRallMWybrowMHermjakobHHuckaMKellDBMüllerWMendesPZellAChaouiyaCSaez-RodriguezJSchreiberFLaibeCDrägerALe NovèreN: Path2Models: large-scale generation of computational models from biochemical pathway maps. *BMC Syst Biol*.2013;7: 10.1186/1752-0509-7-116 116 10.1186/1752-0509-7-116 24180668PMC4228421

[3] ChaouiyaCBérenguierDKeatingSMNaldiAvan IerselMPRodriguezNDrägerABüchelFCokelaerTKowalBWicksBGonçalvesEDorierJPageMMonteiroPTvon KampAXenariosIde JongHHuckaMKlamtSThieffryDLe NovèreNSaez-RodriguezJHelikarT: SBML qualitative models: a model representation format and infrastructure to foster interactions between qualitative modelling formalisms and tools. *BMC Syst Biol*.2013;7: 10.1186/1752-0509-7-135 135 10.1186/1752-0509-7-135 24321545PMC3892043

[4] MüsselCHopfensitzMKestlerHA: BoolNet--an R package for generation, reconstruction and analysis of Boolean networks. *Bioinformatics*.2010;26(10) : 10.1093/bioinformatics/btq124 1378-80 10.1093/bioinformatics/btq124 20378558

[5] KlarnerHStreckASiebertH: PyBoolNet: a python package for the generation, analysis and visualization of boolean networks. *Bioinformatics*.2017;33(5) : 10.1093/bioinformatics/btw682 770-772 10.1093/bioinformatics/btw682 27797783

[6] GargADi CaraAXenariosIMendozaLDe MicheliG: Synchronous versus asynchronous modeling of gene regulatory networks. *Bioinformatics*.2008;24(17) : 10.1093/bioinformatics/btn336 1917-25 10.1093/bioinformatics/btn336 18614585PMC2519162

[7] AlbertIThakarJLiSZhangRAlbertR: Boolean network simulations for life scientists. *Source Code Biol Med*.2008;3: 10.1186/1751-0473-3-16 16 10.1186/1751-0473-3-16 19014577PMC2603008

[8] StollGCaronBViaraEDugourdAZinovyevANaldiAKroemerGBarillotECalzoneL: MaBoSS 2.0: an environment for stochastic Boolean modeling. *Bioinformatics*.2017;33(14) : 10.1093/bioinformatics/btx123 2226-2228 10.1093/bioinformatics/btx123 28881959

# Response

**Published:** 2018-05-04

**Authors:** Amir Rubinstein

**Affiliations:** Tel Aviv University, Israel

Dear Aurélien,

We greatly appreciate the detailed report. Below please find our point-by-point reply. Changes were made to the manuscript in its 2nd version. To ease reading of the responses below, we copy-pasted the comments in the report as well. 

---------------------------------------

Some related work should also be taken into account. In particular Wrzodek et al. ^1^and Büchel et al. ^2^ which generate qualitative models in SBML qual format ^3^ from KEGG pathways. My main concerns are detailed in the following text, none of them would be blocking taken separately, but my overall interpretation is that this work requires deep changes to become convincing.

 

- Regarding the additional resources, they were added to the Introduction. Our focus is at integrating a Cytoscape application (BioNSi) with KEGG, exploiting this convenient simulation environment.

I installed the BioNSi tool in Cytoscape 3.4. The documentation states that building a network from KEGG pathways requires the selection of a folder containing one or several KEGG xml files and a CSV file with expression data. I tried to provide a folder containing two KEGG pathways and an empty CSV file, but it did not work (error message: "number of expression files is not 1"). I had the same problem with Cytoscape 3.6.

 

- In the current version of the tool the csv expression file should not be empty. This is obviously something to fix in the next version. In the meantime, we provide such a file with arbitrary initial levels of 9. The file can be downloaded from the website and from http://www.cs.tau.ac.il/~amirr/files/expression.csv.
- Both Cytoscape 3.4 and 3.6 should work fine.

KEGG pathways often "merge" multiple biological entities in a single node, the paper does not say how this case is handled, the documentation only states that ellipsis dots are removed from the name. Does it mean that these groups are preserved? How does this affect merging multiple KEGG pathways? It can be a concern as the same entity could be involved in different groupings in different KEGG pathways.

 

- BioNSi preserves all the alternative names for a KEGG entity. When merging pathways, even if two nodes have different alternative names, they will be fused into a single node. Furthermore, names of entities in the expression level file are matched against any of the alternative names of a node in the network. This information was emphasized in the new version of the user manual and paper.

More generally, it would make sense to separate the transformation of KEGG pathways into discrete models (taking into account previous work ^1^ ^,^ ^2^), and the merging of multiple discrete models, which is a more general problem, and could be used to integrate several existing models with new models generated from pathway maps.

 

- Such separation of functionality indeed makes a lot of sense. It may be implemented as part of a later version of the tool.  

The import of initial states from experimental data, and in particular the discretization step deserve further discussion. For a generic tool, I suspect that it uses a simple method where the full range of values for all genes is scaled to 0-9, defining 9 evenly-spaced thresholds. Is it the case or are the thresholds based on the range of each gene separately? As mapping experimental data on the model is one of the two core aims of this paper, it should at least review the alternatives, give proper details on how this is handled and state that alternative mapping can be integrated as a preprocessing step.

 

- The initial levels are scaled to 0-9 for all the nodes. The following was added to the paper: "Note that the initial states are scales to 0-9 for the whole network based on the maximal expression level in the network, rather than for the range of each gene separately. Since the scaling depends of the maximal expression level of a node in the network, an extreme outlier may restrict the levels of other nodes to a very limited range. Therefore, the use of logarithmic expression data is recommended, and when the raw data in not logarithmic a simple transformation is required as a preprocessing step."  

Mapping entities in the model to entities in the experimental data is not discussed either, does the tool convert KEGG identifiers to official gene names, does it require the user to do the mapping in advance, does it provide a GUI to fine-tune the mapping afterwards?

 

- We use KEGG xml files which include the complete array of gene\protein names available, including official gene symbol, HGNC id, KEGG id and others. In BioNSi, we use all gene symbols for each entry and therefore, identifiers are not being lost. For the graphical presentation, the first identifier is being used.

The paper does not state what is the objective of using an experimentally measured values as initial state. The experiments presented here seem to represent "steady states" of the biological system, the simulation leads to a steady state which can be compared to the experimentally measured one. Differences between the experimental and computed states can be of two types: (i) inconsistency showing that the model needs further improvement, and (ii) effect that is not measured in the experiment: for example change of phosphorylation which is not measured by expression data. As this is the very core of the paper, a discussion on the possible biological meaning of the results seems required.

- We added such a discussion to the Methods section ( *Simulation*): "We explored two use-cases as a proof of concept, as described in the next section. In both use cases, KEGG pathways were integrated to form a BioNSi network, and gene expression analysis was used as initial nodes' states. In both use cases, the states of nodes changed throughout the simulation process, terminating in a steady state. This change in states may reflect one of two situations: (i) the expression data itself does not represent a biological steady state, and therefore the changes in-silico reflect changes in-vivo, and (ii) data is missing from the network, either structural (missing regulators or connections between existing nodes) or quantitative (weights on edges, initial states, etc.). In the latter case the conclusion is that the model needs further improvement. "  

 

The BioNSi tool, which this work extends, supports simulations of qualitative (discrete) models. Its capabilities are summarized in this paper, and it is briefly compared to other tools for qualitative modelling in Table 1. A minor typo in this table replaced BooleSim with BoolSim (there is also a tool called BoolSim in this field: it is the command line tool handling the Boolean part of SQUAD).

- Fixed.

 

The paper describes BioNSi (and some other tools) as doing synchronous and asynchronous simulations, but misuses the term "asynchronous". Here asynchronous refers to synchronous with delays or priorities (i.e. all transitions do not happen at the same speed, but are still using the same clock), however it usually means independent trajectories, leading to non-deterministic behaviour. Using the classical asynchronous updating of qualitative models, a state can have several separate successors, each corresponding to the update of a single component. This allows to study systems where the relative speeds of transitions is not known, but introduces numerous concurrent trajectories, making the analysis much harder. GINsim is the only tool in the provided list implementing asynchronous updating, it also supports priorities, but not delays. BoolNet ^4^, PyBoolNet ^5^, and Boolsim ^6^ support asynchronous updating. To handle this complexity, some tools such as BooleanNet ^7^ or MaBoSS ^8^ perform random walk in the asynchronous dynamics.

- We changed "asynchronous" to "non-simultaneous", and added this column to Table 1. 

 

In BioNSi, inhibitions have negative weights, while activations have positive weights. These weights are multiplied by the level of their source nodes, the resulting weighted sum is then used to define the evolution of the target element. While this allows to quickly define a model, I am concerned by the lack of flexibility: some inhibitors only block the effect of one of the activators, some regulators have a cooperative effect, some act independently. I agree that the simple weight system used in BioNSi is convenient when these fine-grained mechanisms are unknown: it provides a good first model. However, the KEGG pathways mostly describe processes which have been deeply studied, and for which such an approximation could be avoided. There is some value in a simple system based on weights, especially as it fits better for integration into cytoscape, but building more precise functions through the use of intermediate nodes representing cooperative effects should at least be discussed as a common refinement.

 

- BioNSi (already in the original version) does support additional regulation effects in the form of blocking and enabling edges and external signal nodes. These are only mentioned without details in the end of Box 1. We added a brief description of these in box 1.

Some minor points:

Missing closing of 2 parenthesis on page 2, another on page 3

- We cannot find these missing parenthesis, we would appreciate a clarification.

Page 6: "138 nodes with 8 nodes or less" is confusing. I suspect it means 138 nodes within sub-networks of 8 nodes or less?

- Fixed.

The first paragraph on page 6 ("simulation of different experimental conditions") repeats the last paragraph of the methods.

- We deleted the redundant paragraph from the results.
